# Genetics and Epigenetics: New Insight on Gestational Diabetes Mellitus

**DOI:** 10.3389/fendo.2020.602477

**Published:** 2020-12-01

**Authors:** Maria Grazia Dalfrà, Silvia Burlina, Gloria Giovanna Del Vescovo, Annunziata Lapolla

**Affiliations:** Department of Medicine, University of Padova, Padova, Italy

**Keywords:** obesity, genetic, epigenetic, gestational diabetes, mass spectrometry, nutrition

## Abstract

Gestational diabetes mellitus (GDM) is the most common metabolic complication of pregnancy, with a prevalence that has increased significantly in the last decade, coming to affect 12–18% of all pregnancies. GDM is believed to be the result of a combination of genetic, epigenetic and environmental factors. Following the identification of susceptibility genes for type 2 diabetes by means of genome-wide association studies, an association has also been demonstrated between some type 2 diabetes susceptibility genes and GDM, suggesting a partial similarity of the genetic architecture behind the two forms of diabetes. More recent genome-wide association studies, focusing on maternal metabolism during pregnancy, have demonstrated an overlap in the genes associated with metabolic traits in gravid and non-gravid populations, as well as in genes apparently unique to pregnancy. Epigenetic changes—such as DNA methylation, histone modifications and microRNA gene silencing—have also been identified in GDM patients. Metabolomics has been used to profile the metabolic state of women during pregnancy, based on the measurement of numerous low-molecular-weight metabolites. Measuring amino acids and conventional metabolites has revealed changes in pregnant women with a higher insulin resistance and high blood glucose levels that resemble the changes seen in non-gravid, insulin-resistant populations. This would suggest similarities in the metabolic profiles typical of insulin resistance and hyperglycemia whether individuals are pregnant or not. Future studies combining data obtained using multiple technologies will enable an integrated systems biology approach to maternal metabolism during a pregnancy complicated by GDM. This review highlights the recent knowledge on the impact of genetics and epigenetics in the pathophysiology of GDM and the maternal and fetal complications associated with this pathology condition.

## Introduction

Gestational diabetes mellitus (GDM) is defined as any degree of glucose intolerance developing or first recognized during pregnancy that is not clearly overt diabetes (1). GDM affects from 5–6% to as many as 15–20% of pregnancies worldwide, and its prevalence is increasing due mainly to rising rates of obesity, sedentary lifestyles, and women becoming pregnant at an older age (2, 3). Even if GDM is currently the most common medical complication of pregnancy, no single diagnostic modality to screen and diagnose this condition is universally applied. In this context it is difficult to assess its real frequency and related complications (3, 4).

The increase in placental hormones antagonizing the action of insulin in the second and third trimesters of pregnancy gives rise to a physiological increase in insulin resistance. The purpose of this mechanism is to provide the fetus with glucose, and a gradual increase in insulin secretion enables the woman’s normal glucose levels to be maintained (5). GDM develops when beta cell insulin secretion is unable to compensate for pregnancy-induced insulin resistance, and/or in conjunction with an impaired beta cell function (6–8).

Untreated GDM can lead to adverse outcomes for both the mother and her child during pregnancy and childbirth, including preeclampsia, cesarean delivery, birth trauma, macrosomia, and neonatal hypoglycemia and hyperbilirubinemia (9–11). GDM women have a 2 fold higher risk of developing type 2 diabetes later in life, as well as metabolic syndrome and cardiovascular disease. Furthermore children of these patients are at high risk to develop type 2 diabetes and obesity at an early age (earlier) (9–11). Therefore, greater efforts to reduce obesity and diabetes, especially among girls and young women through lifestyle modifications are necessary (4). Unfortunately, the participation of GDM women in postpartum follow-up programs is still unsatisfactory, emphasizing the importance to improve risk awareness utilizing more proactive patient-contacting attempts (4, 12).

The pathogenesis of GDM is not fully understood, but recent studies have suggested that it could be the result of a combination of genetic, epigenetic and environmental factors.

This review highlights the recent knowledge on the impact of genetics and epigenetics in the pathophysiology of GDM and the maternal and fetal complications associated with this pathology.

## Genetic Studies

Two different approaches have been used to study the role of genes in maternal metabolism during pregnancy. Studies on candidate genes have examined the association between variants of selected genes and the risk of GDM. However, this approach has proved of limited use for the purpose of identifying the genes involved, due to small sample sizes and the inclusion of limited numbers of variants in the genetic loci. Using genome-wide association studies (in which genetic variants across the whole genome are tested for any association with a trait or disease), variants from many different loci have been associated with the risk of GDM. They have each shown only a modest effect, however, so large numbers of individuals need to be assessed to demonstrate a significant association.

Given that GDM, like type 2 diabetes, is characterized by insulin resistance, several genes known to be associated with a higher risk of developing type 2 diabetes have also been investigated in patients with GDM.

Kwak et al. (13). used the genome-wide association approach to compare 1399 women with GDM (cases) and 2025 non-diabetic women (controls). They employed 2,19 million genotyped or imputed markers, and selected 11 loci for further genotyping in stage 2 samples from 931 cases and 783 controls. The effect of genetic variants known to be associated with type 2 diabetes was also investigated in the women with GDM. A significant association emerged between known T2D variants and GDM. This association with GDM was particularly strong for the genetic variants in CDKAL (a type of variant affecting beta cell survival) and MTNR1B (a variant associated with high fasting plasma glucose and insulin levels). These results point to a shared genetic basis for GDM and T2D.

The association between certain genetic variants that raise the risk of T2D and GDM was subsequently studied by Huopio and coworkers in a sample of 533 GDM women and 407 normal pregnant controls, who were genotyped for 69 single-nucleotide polymorphisms (SNPs) (14). Interestingly, the MTNR1B variants rs10830963 and rs1387153 were found significantly associated not only with GDM, but also with impaired fasting plasma glucose levels and some insulin secretion indicators (the Matsuda index, the insulin/glucose AUC, and the AUC adjusted for the Matsuda index) measured in GDM patients after pregnancy (14). A genome-wide association study performed in East Asian population showed that some polimorhisms in KCNQ1 are associated with GDM (15). In 869 Korean GDM women and 632 non diabetic control subjects rs 2074196 and rs 2237892 were associated with the risk of GDM (OR 1.17, 95% CI 1.01–1.36, p=0.039; OR 1.24,95% CI 1.07–1.45, p=0.0049 respectively); furthermore rs 2237892 and rs 2237895 were associated with decreased insulinogenic index at the time of GDM diagnosis (p=0.025 and p=0.037) (15). Ren et al. tested MTNR1B for the association with diabetes related traits in 1.383 subjects at baseline and in 374 subjects over a 3–5 year follow up participating in the family-based Beta Gene study of the Mexican American **(** 16). The rs 10830963 was associated cross-sectionally with fasting glucose (p=0.0069), acute insulin response (AIR, p=0.0013), disposition index (p=0.00078), glucose effectiveness (p=0.018), gestational diabetes (OR 1.48; p=0.012), the rate of change of fasting glucose (p=0.043), No association was found with OGTT 30 min and delta insulin (the difference between the 30 min and fasting glucose concentration), 30 min insulin based disposition index and the rate of change in beta cell compensation for insulin resistance. So the variation in MTNR1B contribute to determine the absolute level but not the rate of changes over time of insulin secretion. The differences in absolute insulin secretion and beta cell compensation could be a factor involved in TD2 and GDM development. This study makes an important contribution to the knowledge of the pathophysiological mechanisms determining type 2 diabetes and GDM.

The insulin receptor substrate 1 (IRS-1) expressed in tissues sensitive to insulin is crucial to glucose transporter 4 translocation (GLUT-4). IRS-1 polymorphism has been found related to insulin resistance, obesity and type 2 diabetes mellitus. In a study on GDM, the frequency of IRS-1 gene polymorphism was significantly higher in women with GDM than in pregnant women with a normal glucose tolerance, suggesting a role for this polymorphism in the onset of GDM as well as type 2 diabetes mellitus (17). The switch on IRS-1 of the amino acid GLY972 Arg (rs1801278) impairs insulin secretion, and a study on 1306 GDM patients and 1973 pregnant women without GDM found a significant association between the presence of this polymorphism and the risk of GDM (18).

Intriguing results were generated by a study on the genetic variants in GCK and TCF7L2, and their possible association with glucose levels on oral glucose tolerance tests (OGTTs) performed in pregnant women participating in the Hyperglycemia and Adverse Pregnancy Outcomes Study (HAPO) (19). This study involved 25000 pregnant women, and was designed to test the association between maternal glycemia and adverse outcomes of pregnancy. The OGTT results showed that the GCK variant was associated with high fasting, 1 h and 2 h glucose levels. In the European women’s offspring, the GCK variant was associated with a high birth weight, fat mass and skinfold thickness as well. Variants in GCK and TCF7L2 were also associated with a higher odds ratio for the onset of GDM (20).

**Table 1** lists the genes responsible for insulin secretion, insulin resistance and glucose metabolism that have been found associated with the risk of GDM (13, 15, 18, 21–26).

**Table 1:** Genes showing an association with Gestational Diabetes Mellitus.

Gene	Chr.	Encoded protein	Protein function	Study Samples size	Limits	Author	
**IRS1**	2	Insulin receptor substrate 1	Substrate of insulin receptor tyrosine kinase; key molecule in the insulin signaling pathway	Meta-analysis 1306 GDM 1973 CONTROLS	Significant heterogeneity among the studies	Zhang et al. (18)	
**IGF2BP2**	3	Insulin-like growth factor 2 mRNA-binding protein 2	Binds insulin-like growth factor 2 mRNA and may regulate protein translation; risk allele associated with decreased insulin secretion	Case-control 931 GDM 783 CONTROLS	Bias of GDM screening in control group	Kwak et al. (13)	
**CDKAL1**	6	CDK5 regulatory subunit associated protein 1 like-1	Function of the protein is not known but not pregnant carriers of the risk alleles have impaired oral and intravenous glucose stimulated insulin secretion	Meta-analysis 2959 GDM 3675 CONTROLS	Only Asian population	Zhang et al. (21)	
**GCK**	7	Glucokinase	Phosphorylates glucose in pancreatic β cells and hepatocytes; involved in the regulation of insulin secretion	Meta-analysis 1934 GDM 6492 CONTROLS	Heterogeneity among the studies	Zhang et al. (21)	
**TCF7L2**	10	Transcription factor 7-like 2	Transcription factor and member of the Wnt signaling pathway; risk allele associated with reduced insulin secretion	Case-control 2636 GDM 6068 CONTROLS	Only gene that were known to be associated with the risk of T2D were studied	Ding et al. (22)	
**MTNR1B**	11	Melatonin receptor 1B	G-protein coupled receptor that is expressed on β cells, binds melatonin and may antagonize insulin release	Meta-analysis 986 GDM 1070 CONTROLS	No indication of bias Heterogeneity among the studies	Zhang et al. (18)	
**KCNJ11**	11	Potassium inwardly rectifying channel, subfamily J, member 11	Integral membrane protein and inward-rectifier type potassium channel that is controlled by G-proteins and associated with the sulphonylurea receptor; involved in the regulation of insulin secretion	Meta-analysis 1837 GDM 4327 CONTROLS	Modest associations	Zhang et al. (21)	
**KCNQ1**	11	Potassium voltage-gated channel, KQT-like subfamily, member 1	Voltage gated potassium channel; involved in the regulation of insulin secretion	Case-control 869 GDM 632 CONTROLS	Only Asian population No normal pregnant women as control group	Kwak et al. (15)	
**GCKR**	2	Glucokinase regulator	Regulatory protein that inhibits glucokinase in liver and pancreatic islet cells	Meta-analysis 3636 GDM 722 CONTROLS	No adjusted for confounding factors	Lin et al. (23)	
**HNF4A**	20	Hepatocyte nuclear factor 4α	Mutation in this gene is associated with MODY	Prospective 5184 GDM 1220 CONTROLS	Only Asian Indian population	Kanthimathi et al. (24)	
**SLC30A8**	8	Solute carrier family 30 member 8	Is expressed only in the pancreas and is related to insulin secretion	Meta-analysis 8204 GDM 15221 CONTROLS	Only Asian population	Lin et al. (23)
**PPARG**	3	Peroxisome proliferator activated receptor γ	Regulator of adipocyte differentiation and glucose homeostasis	Meta-analysis 2517 GDM 3015 CONTROLS	No adjusted for confounding factors Heterogeneity among the studies	Whu et al. (25)
**FTO**	16	Fat mass and obesity associated gene	Involved in regulation of fat mass and adipogenesis and body weight	Case-control 869 GDM 345 FEMALE CONTROLS 287 MALE CONTROLS	No normal pregnant women as control group Only Korean population	Cho et al. (26)

During pregnancy, chronic low-grade inflammation have a role in causing insulin resistance, and several studies have found higher levels of inflammatory mediators in GDM patients and their newborn (27, 28). Using a candidate gene approach, some metabolism indicators and variants in 31 inflammatory pathway genes were examined in women taking part in the HAPO study. A significant association emerged between some maternal metabolism indicators (fasting and 1 h plasma glucose, C-peptide, HbA1c) and six of the genes investigated, suggesting that inflammation would contribute to the metabolic phenotype in pregnancy (**Table 2**) (29).

**Table 2:** Inflammatory pathway genes associated with maternal metabolism (29).

Gene	Encoded protein	Chromosome	Trait
**LEPR**	Leptin receptor	1p31.2	1-h C-peptide
**IL8**	Interleukin 8 receptor	4q13.3	1-h plasma glucose
**TNFα**	Tumor necrosis factor α	6p21.33	HbA1c
**IL6**	Interleukin 6	7p15.3	1-h plasma glucose
**ADIPOR2**	Adiponectin receptor 2	12p13.33	Fasting C-peptide
**RETN**	Resistin	19p13.2	Fasting plasma glucose

In order to evaluate the effect of preexisting maternal obesity and gestational diabetes on the expression of genes involved in adipokines regulation and lipid metabolism, human subcutaneous adipose tissues were analyzed by quantitative RT-PCR. The gene expression of the adipokines TNFalfa, IL-1beta, and leptin were increased in adipose tissue from obese (No.10) and GDM (No. 18) with respect to normal control women (No. 28). Furthermore, decreased gene expression was found for some transcription factors involved in lipid metabolism as LXRα, PPARα, PPARδ, PPARγ, RXRα and SREBP1c. These data underline the importance of the excess of substrate supply from the mother to the fetus in determining neonatal adiposity in obese and GDM pregnant women (30).

The genetic studies published until now show that several genes are involved in the physiophatology of GDM. However, the most of the studies have been conducted only in some populations (e.g. Asia, Korea) and this could explain the reason why they are not reproducible across population; furthermore, some studies are not adjusted for confounders (maternal age, diet, scolarity). Studies linking genetic variants to environmental factors are needed to understand the physiophatology of GDM.

## Epigenetic Studies

Epigenetics is the study of changes in gene expression or phenotype prompted by mechanisms other than variations in DNA sequences. Examples of epigenetic changes include methylation, histone modifications, and messenger RNA (mRNA) binding by microRNAs (miRNAs).

Genes react to a pregnant woman’s metabolic status *via* epigenetic mechanisms, and studies have shown that even a slight increase in glycemia can be associated with epigenetic adaptations *via* the so-called “metabolic memory”. It is worth emphasizing here that metabolic memory does not only concern what happens in diabetic patients in the short term. It also has to do with long-term metabolic effects in the developing uterine environment. The concept of metabolic memory fits well with what Barker hypothesized in the 1980s about individuals being programmed to cope with nutritional thrift in intrauterine life in order to survive the damage caused by poor or over- nutrition after birth (31). Several studies have examined the association between methylation and GDM. Wu and coworkers described genome-wide DNA methylation changes in pregnant women’s blood before any GDM was diagnosed. They identified a series of differently methylated genes that the woman’s blood, umbilical cord and placenta shared, i.e. Hook Microtubule Tethering Protein 2 (HOOK2), Retinol Dehydrogenase 12 (RDH12), Constitutive photomorphogenic homolog subunit 8 (COPS8), Phosphoinositide-3-kinase, regulatory subunit 5(PIK3R5), 3-hydroxyanthranilate 3,4-dioxygenase (HAAO), Coiled-coil domain containing 124 (CCDC 124) and Chromosome 5 open reading frame 34 (C5orf 34). HOOK2 codes for a protein that mediates binding to organelles, and is involved in cilia morphogenesis and endocytosis. RDH12 encodes a retinal reductase involved in short-chain aldehyde metabolism. COPS8 is a regulator of multiple signaling pathways. PIK3R5 is involved in cell growth, proliferation, differentation, motility, survival and intracellular trafficking. HAAO catalyzes the sintesis of quinolonic acid (QUIN), which is an excitotoxin that may partecipate in the pathogenesis of neurologic and inflammatory disorders. CCDC 124 is involved in cell cycle and cell division, C5orf 34 is unknown, but sequence is conserved in chimpanzee, Rhesus monkey, dog, cow, mouse, rat, chicken and zebrafish (32).

Cardenas et al. (33) conducted an epigenome-wide association study (involving 850000 CpG sites) on samples of placenta and plasma glucose, correlating them with 2 h post-OGTT plasma glucose levels in 448 mother-and-infant pairs at 24–30 weeks of gestation. They found plasma glucose at 2 h OGTT positively associated with a lower DNA methylation of 4 CpG sites within the phosphodiesterase 4b gene. Then 3 other CpG sites in the TNFRSF1B, LDLR and BLM genes were found differentially methylated with respect to maternal glucose. TNFRSF1B is involved in apoptosis, the inhibition of cell fusion, and epithelial shedding. LDLR encodes a lipoprotein receptor that mediates LDL endocytosis in the cells, and is also expressed in the placenta. BLM is associated with genome stability and maintenance. When DNA methylation was correlated with the expression of its respective genes in placental tissue, it emerged that maternal glycemic levels during pregnancy were associated with placental DNA methylation of inflammatory genes, the expression of which depends on epigenetic changes. Bouchard and coworkers (34) found that lower DNA methylation levels in the ADIPOQ promoter on the fetal side of the placenta correlated with higher maternal glucose levels during the second trimester of pregnancy. Lower DNA methylation levels on the maternal side of the placenta were associated with a higher insulin resistance, according to the Homeostasis Model Assessment Method (HOMA), during the second and third trimesters of pregnancy; and higher circulating adiponectin levels throughout pregnancy. Given the insulin-sensitizing properties of adiponectin, the latter epigenetic modifications could prompt changes in glucose metabolism in the mother and offspring later in life.

A number of studies sought a link between epigenetic changes in GDM and the newborn’s development. Hajj et al. (35) tested the effect of GDM on the epigenome of the women’s offspring, analyzing cord blood and placental tissue from the newborn of women with GDM, 88 of them treated with dietary measures and 98 with insulin. The maternal imprinting MEST gene and the non-imprinting glucocorticoid receptor NR3C1 gene (both of which are associated with placental and fetal growth) showed significantly lower methylation levels in both tissues in both GDM groups compared with pregnant women without GDM. MEST methylation was also found significantly lower in the blood of adults with obesity than in normal-weight controls. These results indicate that intrauterine exposure to GDM has lasting effects on the epigenome of the offspring, and that epigenetic malprogramming of MEST can contribute to predisposing individuals to obesity later in life. Another study compared genome-wide methylation patterns in fetal cord blood between pregnant women with and without GDM. Using Illumina 450K methylation arrays, significant differences in methylation emerged between GDM patients and control pregnant women. These differences were more evident in GDM women on insulin than GDM on diet therapy and could be explained by more severe episodes of hypeglycemia in GDM requiring insulin treatment. Changes in methylation were seen for: ATPSA1, which encodes a subunit of mitochondrial ATP synthetase that prevents mitochondrial oxidation; MFAP4, which is involved in cell adhesion and intercellular interaction; PRKCH, which belongs to the protein C family and is involved in various signaling pathways; and SLC17A, an intestinal sodium/phosphate cotransporter associated with hypoxia. Interestingly, all these changes in methylation had a small effect size, but affected multiple genes/loci (36) (**Table 3**). Recently, methylation profiles were measured in peripheral blood of 93 GDM offspring and 945 controls from the Danish National Birth Cohort, aged 9–16 years, using an illumine Human Methylation 450 Bead Chip (37). Seventy six differentially methylated GpGs in GDM offspring compared to controls were identified (FDR P<0.05). Adjusting for offspring BMI did not change the association with GDM, even if most of the epigenetic changes resulted associated with maternal BMI, 13 methylated genes were independently associated with maternal GDM. This study supports the close association between GDM and pre-pregnancy BMI and strongly suggests that this association has an effect on offspring epigenetic profile involved in fetal metabolic programming.

**Table 3:** Gene methylation in Gestational Diabetes Mellitus.

Gene	Protein encoded	Function	Study Samples size	Limits	Author
**COPS8**	Constitutive photomorphogenic homolog subunit 8	Regulator of multiple signaling pathways	Case control 11 GDM 11 CONTROLS	Small sample size	Wu et al. (32)
**PIK3R5**	Phosphoinositide-3-kinase, regulatory subunit 5	Cell growth, proliferation, differentiation, motility, survival, and intracellular trafficking			
**HAAO**	3-hydroxyanthranilate 3,4-dioxygenase	Catalyzes the synthesis of quinolonic acid (QUIN), which is an excitotoxin that may participate in the pathogenesis of neurologic and inflammatory disorders			
**CCDC124**	Coiled-coil domain containing 124	Cell cycle, cell division			
**C5ORF34**	Chromosome 5 open reading frame 34	Unknown, but sequence is conserved in chimpanzee, Rhesus monkey, dog, cow, mouse, rat, chicken, and zebrafish			
**HOOK2**	Hook microtubule tethering linker protein 2	Protein that mediates binding to organelles involved in morphogenesis of ciglia and endocytosis			
**RDH12**	Retinol reductase	Protein involved in metabolism of short-chain aldeydes			
**TNFRSF1B**	TNF receptor superfamily member 1B	This protein and TNF-receptor 1 form a heterocomplex that mediates the recruitment of two anti-apoptic proteins: c-IAP1 and c-IAP2, which possess E3 ubiquitin ligase activity	Prospective 448 WOMEN	Correlation with plasma miRNA was not evaluated	Cardenas et al. (33)
**LDLR**	Low density lipoprotein receptor	LDLR pick up LDLs circulating in the bloodstream and transport them into the cell.			
**BLM**	Bloom syndrome protein	Member of a protein family called RecQ helicases			
**PDE4B**	Phosphodiesterase 4B	Regulator of cellular concentrations of cyclic nucleotides and thereby play a role in signal transduction.			
**ADIPOQ**	Adiponectin	Insulin sensitizing, antiinflammatory functions	Prospective 98 WOMEN	Correlation with neonatal outcomes was not evaluated	Bouchard et al. (34)
**MEST**	Encodes a member of αβ hydrolase superfamily	Regulation of adipocyte size and fat mass expansion	Prospective 88 diet treated GDM 98 insulin treated GDM 65 CONTROLS	Small sample size Correlation with neonatal outcomes was not evaluated	Hajj et al. (35)
**NR3C1**	Glucocorticoid receptor protein	Maternal fetal exchange of circulating glucocorticoids			
**ATPSAI**	A subunit of mitochondrial ATP synthetase	Prevention of mithocondrial oxidation	Prospective 88 diet treated GDM 105 insulin treated GDM 120 CONTROLS	Correlation with neonatal outcomes was not evaluated	Haerle et al. (36)
**MFAP4**	Microfibrillar associated protein 4	Cell adhesion and intracellular interactions			
**PRKCH**	Protein kinase C family	Involved in cellular signaling pathways			
**SLC17A**	Intestinal sodium/phosphate cotransporter protein	Mediates transmembrane cotrasport of Na/P in oocites			
**H3K27/H3K4**	DNA histone3 packaging protein	Histone involved in structure of chromatin in cells	Prospective 8 GDM not developing post-partum T2D 6 GDM developing post-partum T2D 6 preexisting T2D 7 CONTROLS	Small sample size	Michalczyk et al. (37)

Women with a history of GDM are at greater risk of developing type 2 diabetes after pregnancy. With this in mind, Michalczyk et al. (38) analyzed several epigenetic markers during and after pregnancy in: women with GDM who subsequently developed type 2 diabetes (No. 6); women with GDM who did not develop type 2 diabetes after delivery (No. 8); women with type 2 diabetes (No. 6); and non-diabetic women (No. 7). When the Authors calculated the proportion of total H3 histone that was methylated, it was: 50% lower for H3K27 at 8–10 and 20 weeks post-partum in the women with GDM who developed type 2 diabetes after pregnancy than in the non-diabetic women; and 75% lower for H3K4 at 8–10 weeks post-partum in the women with GDM who developed type 2 diabetes after pregnancy than in the women with GDM who did not. Although it was conducted on a small sample (No. 27), this study demonstrated that the proportion of histone methylation could be a useful predictor of type 2 diabetes in women with GDM.

Thus DNA methylation could be considered an interesting diagnostic and prognostic marker. However, the most of the studies published in the frame of GDM have limited sample size, do not correlate the markers with maternal and/or neonatal outcomes, furthermore different methods to measure DNA methylation have been utilized. These limitations make difficult to generalize the meaning of the studies.

MiRNAs are small non-coding RNAs that regulate gene expression and various cell functions by directing messenger RNA degradation or inhibiting its translation (39) (**Table 3**).

Some recently-identified miRNAs have been associated with insulin secretion, insulin resistance, and inflammation, and differences have emerged in some circulating miRNA levels between individuals with and without type 2 diabetes (40). Zhao and others (41) examined some miRNAs in pregnant women at 16–19 weeks of gestation (WG), finding a significantly lower expression of 3 miRNAs (miR-29a, miR-132 and miR222) in women who went on to develop GDM at 24-28 WG than in those who did not develop GDM. MiR-29 plays a part in glucose homeostasis: its overexpression inhibits insulin-stimulated glucose uptake and downregulates gluconeogenesis (42). MiR-132 targets the insulin-mediated regulation of cytochrome P450 (which is involved in hepatic metabolism), and it has a role in trophoblast expansion (its reduced expression impairs normal trophoblast development) (42, 43). MiR-222 is involved in regulating the cell cycle (controlling the cyclin-dependent kinase inhibitor).

Cao and coworkers (44) found miR16.5, miR17-5, and miR20a-5p the best predictors of GDM, with a ROC curve of 0.92, 0.88, and 0.74, respectively. MiR16.5 is involved in a series of processes related to insulin resistance, and it is upregulated in type 2 diabetes. MiR17-5 is involved in smooth muscle cell proliferation, and plays a part in the vascular complications of diabetes. MiR20a-5p is upregulated in preeclampsia, a condition often related to GDM.

Wander et al. (45). examined miRNA levels in pregnant women with GDM in an effort to see whether body mass index had any influence on its onset. MiR155-5p, and 21-3p were found positively associated with GDM, and an association between this condition and miR21-3p, and miR210-3p was only seen in overweight/obese women. MiR-155 and MiR21-3 have a role in pathways that regulate cell survival, such as apoptosis, cell cycle regulation, and response to inflammation. MiR210-3p is associated with angiogenesis (43).

Cao and coworkers looked into the role of miR-98 in placental tissues from GDM patients (46). MiR-98 is upregulated in the kidney in type 2 diabetes, and it is involved in embryo implantation. The results of the study indicate that miR-98 is upregulated and total DNA methylation levels are reduced in the placentas of GDM patients by comparison with those of normal pregnant women. MiR-98 targets Mecp2 and also regulates the Mecp2 target gene, and this could have important consequences for the fetal growth (**Table 4**).

**Table 4:** Studies assessing the role of mRNAs in gestational diabetes (GDM) and Normal Glucose Tolerance (NGT).

miRNA	Number of case	miRNA impaired	Significance	Limits	Author
**miR-132**	58 GDM-50 NGT	Reduced expression	Insulin mediated regulation of cytocrome P450	Small sample size Only Chinese population	Zhou et al. (42)
**miR-132**	24GDM-24NGT	Reduced expression	Insulin mediated regulation of cytocrome P450	Small sample size Only Chinese population	Zhao et al. (41)
**miR29a**	24GDM-24NGT	Reduced expression	Glucose homeostasis		
**miR222**	24GDM-24NGT	Reduced expression	Regulation of cell cycle		
**miR16-5p**	85GDM-72NGT	Up-regulation	Process related to insulin sensitivity	Only candidate genes studied	Cao et al. (44)
**miR17-5p**	85GDM-72NGT	Up-regulation	Smooth muscle cell proliferation		
**miR20a-5p**	85GDM-72NGT	Up-regulation	Regulator cell activity and ROS levels		
**miR 155-5p**	36GDM-80 NGT	Overexpression	Regulation of cell survival as apoptosis and response to inflammation	Only candidate genes studied Most of patients were Hispanic Few cases of GDM	Wander et al. (45)
**miR 21-3p**	36GDM-80 NGT	Overexpression	Regulation of cell survival as apoptosis and response to inflammation		
**miR210-3p**	36GDM-80 NGT	Overexpressiion	Regulation of angiogenesis		
**miR98**	193GDM-202NGT placenta samples	Up-regulation	Embrio implantation	Only Chinese population	Cao et al. (46)

Recent years have seen a few studies conducted to identify specific miRNAs as predictors of GDM, and of maternal and fetal outcomes. The results have been promising, but inconclusive for the time being. Further studies need to be performed, bearing in mind that miRNA expression in pregnancy varies with weeks of gestation, the sex of the fetus, the type of sample considered (blood, placenta, cord blood, and so on), and the mode and type of delivery (47).

## New Methodological Approaches

Metabolomics is a relatively new-omic technology capable of shedding light on an individual’s metabolic status by measuring their low-molecular-weight metabolites, almost 4000 of which have been identified in human serum (48). Their identification was made possible thanks to the use of highly precise and accurate methods, such as magnetic resonance spectroscopy and mass spectrometry (49, 50).

In a study using magnetic resonance spectroscopy, the levels of some urinary metabolites—such as 3-hydroxyisovaleriate, 2-hydroxybutyrate, and choline—were found higher in GDM patients than in pregnant women without GDM (51). High 3-hydroxyisovaleriate levels reflect a reduction in biotin status. Levels of 2-hydroxybutyrate are reportedly associated with insulin resistance, and have been proposed as markers of the onset of type 2 diabetes (52).

Liu et al. (53) combined metabolic and genetic data relating to insulin sensitivity during pregnancy obtained from samples collected from women participating in the HAPO study, who underwent OGTT at 24–28 WG. Data on metabolites were obtained using targeted and untargeted mass spectrometric approaches, and the genetic information came from a genome-wide association study. Several metabolites were found associated with maternal insulin sensitivity, including amino acids, lipid metabolites, fatty acids and carbohydrates. The genome-wide association study identified 12 genetic variants in the GCKR locus significantly associated with insulin sensitivity, as measured with the insulin sensitivity index. Then the common GCKR variant rs1260326 was found significantly associated with a number of fasting and 1 h post-OGTT concentrations of metabolites such as triacylglycerol, 2-hydroxybutyrate, lactate, 2-ketoleucine, and palmitoleic acid. Mediation analysis suggested that 1 h palmitoleic acid concentrations contribute to the association between rs1260326 and insulin resistance. This study identified associations between metabolites, genetic markers and insulin sensitivity in pregnant women, shedding new light on the pathophysiology of GDM. A comprehensive analysis of metabolomic studies performed in China evaluating by mass spectrometry a series of urinary metabolites in GDM women has proposed a new view in the relationship between obesity and GDM (54). In particular the author propose that obesity determines the production of proinflammatory molecules that activates the tryptophan-kynurenine pathway and xanturenic acid synthesis so determining hyperglycemia. Hyperglycemia in its turn determines an increase of the nucleotide and acid uric synthesis. Supeoxide anions are produced as byproducts. The increased production of xanthurenic acid, uric acid and superoxide anions contributes to the development of GDM.

Proteomics (55) is an approach that uses mass spectrometry to identify any changes in protein expression due—in the case of interest here—to hyperglycemia and GDM. The method has been used mainly to study the placenta (56–60). It is well known that the placenta undergoes a number of structural and functional changes in diabetes because of the abnormal maternal milieu brought on by high glucose and insulin levels, which increase the production of inflammatory cytokines (56–58). In this setting, applying different mass spectrometry approaches—such as MALDI-MS and LC-MS^E^—to the study of placental samples from women with and without GDM generated evidence to show that, if well controlled, GDM induces only minor changes in the placental proteome (59, 60).

The studies published until now show differences in the metabolic profiles of mothers with GDM with respect to pregnant non diabetic women. This finding need to be confirmed by large studies that should also evaluate the relationships between the changes in metabolic profile and fetal outcomes.

## Nutrigenetics in GDM

Nutrigenetics is the study of the effect of genetic variation on dietary response and of the implication of these interactions on the health status and on the diseases related to nutrition (61).

Recent evidence suggests that dietary factors can induce epigenetic changes, that in turn can affect genome stability and the expression of miRNAs and proteins involved in metabolism (62).

In a study that investigated the association between some clinical parameters and nine single nucleotide polymorphisms (SNPs) involved in nutrition and metabolism in women with and without GDM, the TCF7L2 gene variant rs7903146 showed a strong association with the risk of developing GDM (OR: 2.56; 95% CI: 1.24–5.29). A positive correlation was found between lipid parameters and polymorphisms of PPARG2 (p=0.03), APOA5 (p=0.02), MC4R (p=0.03), LDLR (p=0.04) and FTO (p=0.03). The rs17782313 variant, close to the MC4R gene, was associated with pre-pregnancy BMI (p=0.02) in the women with GDM. The significant associations between the GDM women’s nutritional parameters and some gene variants involved in nutrition and metabolism could be key to the development of effective tools to prevent GDM (63). In another study aiming to assess the role of novel nutrigenetic markers, alongside traditional parameters, in predicting early, subclinical atherosclerosis in 29 women with a history of GDM, the Authors measured 9 SNPs from 9 genes related to nutrition and metabolism, which were genotyped using high-resolution melting analysis. During a 3-year postpartum follow-up, all the women had an OGTT, their carotid artery intima-media thickness (cIMT) was measured, and their metabolic parameters were analyzed. Significant associations were found between the APOA5-CC genotype and cIMT, and between the CC-APOA5/CC-LDLR interaction and cIMT. This preliminary evidence suggests a role for lipid profile during pregnancy and some genetic variants in predicting cIMT, which is an early marker of cardiovascular disease (64).

**Figure 1:**
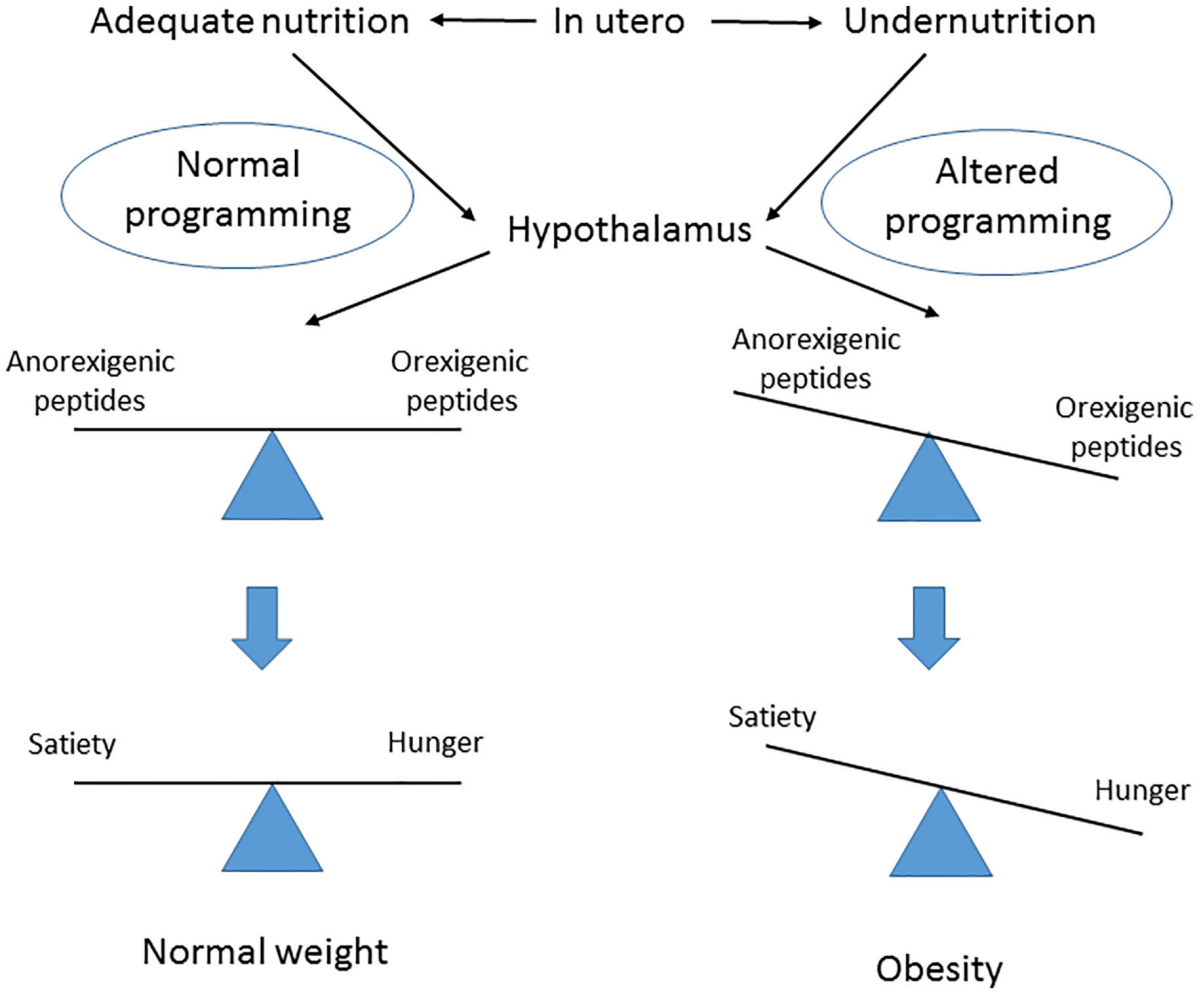
Postulated effect of fetal nutrition in utero on hypothalamus central regulator of obesity programming and subsequent risk later in life.

Eating habits are a key factor influencing human health and fertility. A fetus’s adequate nutrition in utero ensures a normal hypothalamic programming with a balanced production of anorexigenic and orexigenic peptides, and this gives rise to normal feeding patterns in adult life. If a pregnant woman is not adequately nourished, an altered hypothalamic function develops in her offspring, with an exacerbation of the orexigenic state that causes hyperphagia, reduced satiety and metabolic syndrome later in life (**Figure 1**). In particular it has been shown that after periods of famine such as those of Dutch (65), China (66) and Austria (67) offspring born during and after show an increased risk to develop metabolic syndrome. Interestingly, in the exposed children of the Duch famine low levels of DNA methylation of the imprinting IGF2 gene have been reported (68). On the other side studies on rodents and primates mothers nourished with high fat diet determined insulin resistance, high blood pressure and metabolic syndrome in the offspring (69) due to epigenetic modifications of leptin and adiponectin gene expression (70).

**Figure 2:**
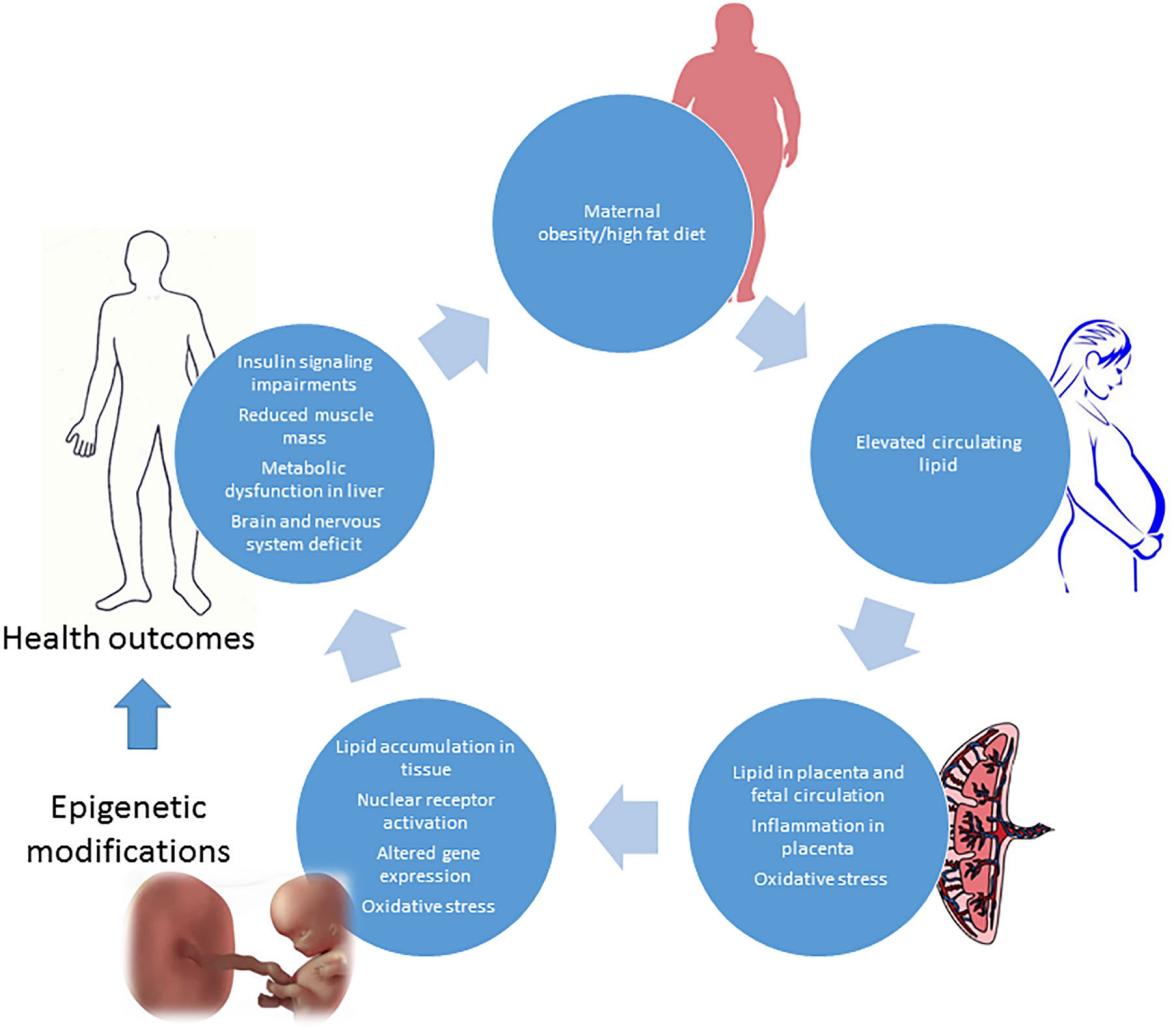
Obesity and high fat diet in pregnancy: impact on fetus development and adverse health outcomes in adult later in life.

As for the offspring of women with GDM, an excessive intake of food during gestation and consequent excessive weight gain give rise to a lower adiponectin expression, higher leptin expression, and hypermethylation of adiponectin in adipose tissue of the fetus (71). A diet rich in lipids during gestation also induces hypermethylation in the offspring’s liver (72). These adverse effects carry a trangenerational risk of metabolic syndrome in the fetus that persists for three generations, even if subsequent pregnancies are characterized by a normal diet (73) (**Figure 2**).

In short, a correct lifestyle and diet are essential to avoid the negative effects of genetic and epigenetic changes. A study on rat pancreatic islets showed that the transcription factor Hhnf4a, which is involved in the onset of type 2 diabetes, is epigenetically regulated by a pregnant woman’s diet, establishing a link between mother nutrition and long-term health of offspring (74). Diet and exercise in obese fathers were able to recover an appropriate adiposity and insulin sensitivity in their female offspring (75). Finally, a protective role of bioactive dietary compounds—in terms of reducing epigenetic changes—has recently been suggested (76).

The connection of these genomic information along with high-specific “omic” technologies will enable the acquisition of new knowledge in order to obtain a better understanding of nutrient-gene interactions depending on the genotype and to develop personalized nutrition strategies for optimal health and disease prevention.

## Conclusions

Interactions between genetic, epigenetic and environmental factors are a key factor in the onset of GDM. Genome-wide association studies first demonstrated that some T2D susceptibility genes are associated with GDM too, suggesting that the genetic architecture of type 2 diabetes and GDM are similar to some degree. Then more recent studies revealed that some genes are unique to pregnancy. Epigenetic changes, such as DNA methylation, histone modifications, and miRNA gene silencing, have also been identified in women with GDM. Study limitations are the small sample size, ethnic differences, and related type of foods. Larger studies, that include different ethnic group to differentiate the impact of ethnicity, lifestyle and nutritional habits on genetics/epigenetics expression are needed. For clinical application of epigenetic and genetic data it is necessary to reduce the number of genes involved to be analyzed. If it were possible to identify a limited number of measurable genes they could be considered in screening and diagnosis of GDM. As regards, on the other side, the relationship with maternal and fetal outcomes no data is available yet. Actually, in clinical practice, we can only give nutritional and lifestyle indications to GDM women in order to reduce the impact of genetics/epigenetics on mother and child health, considering that both nutrition and physical activity showed a positive effect on genetic/epigenetic modification. Future studies combining data from multiple technologies will give us a deeper understanding of the pathophysiology behind GDM. The roles of dietary patterns, nutrients, bioactive substances, and exercise in affecting the genome and epigenome need to be clarified, so that they can be used effectively and appropriately in cases of GDM to reduce the medium- and long-term complications for the mothers and their offspring.

## Author Contributions

AL wrote the manuscript with support from MD, SB, and GD. MD and SB handled the reference resource. MD and GD realized tables and images. All authors contributed to the article and approved the submitted version.

## Conflict of Interest

The authors declare that the research was conducted in the absence of any commercial or financial relationships that could be construed as a potential conflict of interest.
